# Protein- and Peptide-Based Virus Inactivators: Inactivating Viruses Before Their Entry Into Cells

**DOI:** 10.3389/fmicb.2020.01063

**Published:** 2020-05-25

**Authors:** Xiaojie Su, Qian Wang, Yumei Wen, Shibo Jiang, Lu Lu

**Affiliations:** ^1^Key Laboratory of Medical Molecular Virology (MOE/NHC/CAMS), School of Basic Medical Sciences, Fudan University, Shanghai, China; ^2^Lindsley F. Kimball Research Institute, New York Blood Center, New York, NY, United States

**Keywords:** enveloped virus, envelope proteins, inactivation, virus inactivator, emerging viruses

## Abstract

Infectious diseases caused by human immunodeficiency virus (HIV) and other highly pathogenic enveloped viruses, have threatened the global public health. Most antiviral drugs act as passive defenders to inhibit viral replication inside the cell, while a few of them function as gate keepers to combat viruses outside the cell, including fusion inhibitors, e.g., enfuvirtide, and receptor antagonists, e.g., maraviroc, as well as virus inactivators (including attachment inhibitors). Different from fusion inhibitors and receptor antagonists that must act in the presence of target cells, virus inactivators can actively inactivate cell-free virions in the blood, through interaction with one or more sites in the envelope glycoproteins (Envs) on virions. Notably, a number of protein- and peptide-based virus inactivators (PPVIs) under development are expected to have a better utilization rate than the current antiviral drugs and be safer for *in vivo* human application than the chemical-based virus inactivators. Here we have highlighted recent progress in developing PPVIs against several important enveloped viruses, including HIV, influenza virus, Zika virus (ZIKV), dengue virus (DENV), and herpes simplex virus (HSV), and the potential use of PPVIs for urgent treatment of infection by newly emerging or re-emerging viruses.

## Introduction

Human immunodeficiency virus (HIV), influenza virus and many other viruses are enveloped viruses (Harrison, 2008). In most cases, viral envelope derives from the host cell membrane, while in some cases, it derives from the organelle membrane. For example, the envelope of both DENV and ZIKV derives from the endoplasmic reticulum membrane (Kuhn et al., 2002). One or more envelope glycoproteins (Envs) are expressed on the surface of the viral envelope and can play important roles in viral entry into the target cell, including attaching the virus to surface receptor of the host cell and mediating virus-cell membrane fusion, allowing the viral genome to enter the host cell for replication (Vigant et al., 2015; Lu et al., 2016).

**FIGURE 1:**
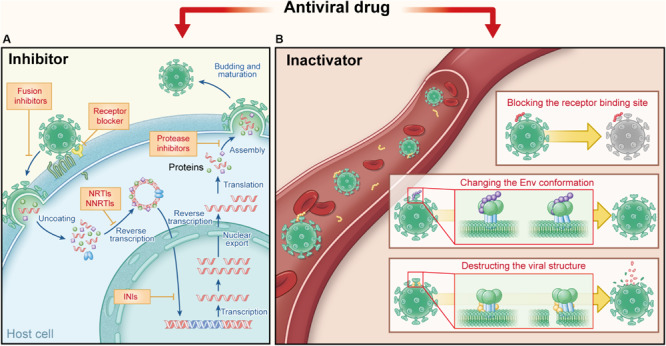
Comparison of current antiviral drugs and the protein- and peptide-based inactivators, taking HIV as the example. **(A)** Major targets and mechanisms of the current anti-HIV drugs. They must act in the presence of target cells: receptor antagonists bind the cell surface receptor to prevent the virus binding and fusion inhibitors target the fusion process, while other inhibitors target viral replication in HIV-infected cells, including nucleoside transcriptase inhibitors (NRTIs) and nucleoside transcriptase inhibitors (NNRTIs), integrase inhibitors (INIs), and protease inhibitors (PIs). **(B)** Targets and action mechanisms of the protein- and peptide-based HIV inactivators. They can actively attack and inactivate cell-free virions that they meet in the blood, by blocking the RBS on viral Envs, changing the Env conformation or destructing the viral structure.

The outbreak of infectious diseases caused by highly pathogenic enveloped viruses, such as HIV, has posed threat to public health worldwide. Thus, it is essential to develop safe and effective antiviral drugs to combat these infectious diseases (Wang et al., 2017). Since the first antiviral drug for treatment of HIV infection, zidovudine, was approved for clinical use in 1987, the United States FDA has approved >80 antiviral drugs so far. They are mainly applied to treat and prevent infection of HIV, herpes simplex virus (HSV), influenza virus, hepatitis B virus (HBV), and hepatitis C virus (HCV) (Chaudhuri et al., 2018). However, most of these antiviral drugs are considered as “passive defenders” because they have to enter the virus-infected cells to inhibit viral replication in the cells (Figure 1A). Therefore, these drugs must possess the ability to penetrate the cell membranes without affecting the normal function of the intracellular proteins. Consequently, they have the weakness of relatively low utilization rate because most part of a drug remaining outside the infected cells does not participate in inhibition of viral infection.

In contrast to “passive defenders,” some antiviral agents used in clinics or under development serve as “gate keepers” to combat viruses outside cells. As mentioned above, viral entry can be divided into two steps: viral attachment to cell receptor(s) and then viral fusion with the target cell. Therefore, “gate keepers” can be classified into three groups: attachment inhibitors that inhibiting the attachment of virions to the target cell by blocking binding of viral Env (e.g., gp120) to cellular the receptor (e.g., soluble CD4) (Deen et al., 1988), receptor antagonists that bind to the cell surface receptor to prevent binding of virions to the receptor (e.g., maraviroc) (Fatkenheuer et al., 2005), and fusion inhibitors that inhibit fusion between viral and target cell membranes (e.g., enfuvirtide) (Jiang et al., 1993a, b; Wild et al., 1994; Lalezari et al., 2003). In general, attachment inhibitors possess some virus inactivation abilities in mechanism, more or less, due to their ability to block the receptor-binding site (RBS) on viral Envs (Lu et al., 2012; Qi et al., 2017). Therefore, attachment inhibitors belong to virus inactivators here; and “gate keepers” includes virus inactivators, receptor antagonists, and fusion inhibitors (Figure 1A).

Different from fusion inhibitors and receptor antagonist blockers that must act in the presence of target cells, virus inactivators can actively attack and inactivate cell-free virions in the blood, through interaction with one or more sites in Envs on virions. The mechanisms of virus inactivators vary: they can bind and block the RBS on viral Envs (Chen et al., 2014), or induce the conformational change of Env, causing virions to lose the ability to enter the host cell (Lu et al., 2012). Some other inactivators may bind to the Env stem or the viral lipid membrane, to disrupt the integrity of the viral envelope or lead to the release of viral genetic materials (Yu et al., 2017) (Figure 1B). Because they can actively attack and then inactivate cell-free virions anywhere they meet in the blood, they should have higher utilization rate than the current antiviral drugs. They are expected to be much safer for *in vivo* human application than the chemical-based virus inactivators (e.g., detergents), most of which can non-specifically lyse lipid membranes of viruses and cells (Polsky et al., 1988; Phillips et al., 2000). PPVIs also have potential for further development as novel antiviral drugs for the urgent treatment of infection by the highly pathogenic emerging and re-emerging viruses.

In this review, we focus on an update of recent developments of PPVIs against several important enveloped viruses, including HIV, ZIKV, influenza virus, DENV, and HSV, and their mechanisms of action. We have also discussed their advantages and disadvantages, compared with the traditional antiviral drugs and the potential application for urgent treatment of infection by newly emerging and re-emerging viruses.

### Protein- and Peptide-Based HIV Inactivators

Human immunodeficiency virus primarily targets the immune system, including CD4^+^ T cells and macrophages. After sexual transmission, HIV enters into CD4^+^ cells in the mucosal tissues and then spreads to the lymphoid organs within days (Haase, 2005; Moir et al., 2011). The immune system of the HIV-infected patient is gradually destroyed, eventually resulting in acquired immunodeficiency syndrome (AIDS) and death (Moir et al., 2011). More than 40 anti-HIV drugs have been approved by the United States FDA, most of which are reverse transcriptase inhibitors (RTIs, including NRTIs and NNRTIs), protease inhibitors (PIs) and integrase inhibitors (INIs) (Deeks et al., 2015). They must enter HIV-infected cells to inhibit viral replication. The only peptide-based HIV fusion inhibitor, enfuvirtide (also known as T20) (Jiang et al., 1993a; Wild et al., 1994; Lalezari et al., 2003), and a small-molecule CCR5 antagonist, maraviroc (Fatkenheuer et al., 2005), must act on the cell surface where the virus binds to the cellular receptor (Lu et al., 2016). These drugs have shown good effects in combating HIV; however, they cannot attack the cell-free virions in the blood, thus also having the problem of low utilization rate.

**FIGURE 2:**
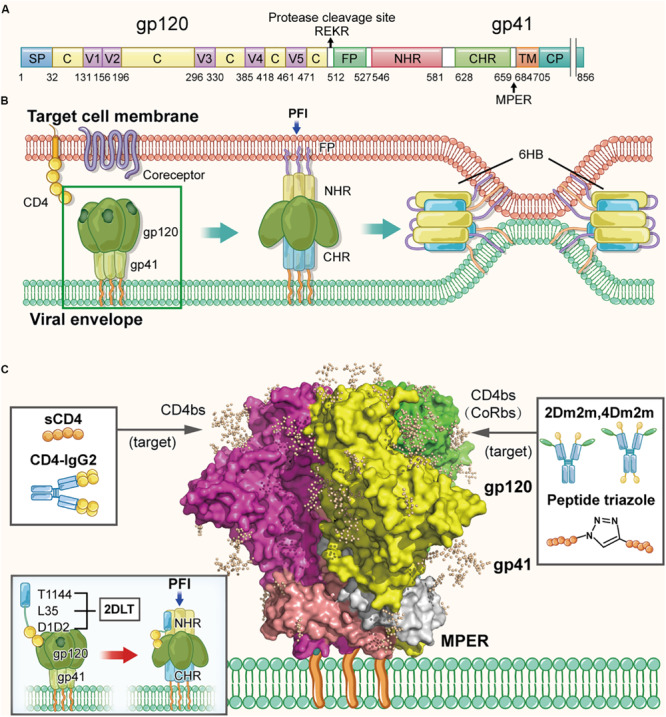
Structure of HIV-1 Env in the native and fusion-intermediate states, which serve as targets for protein- and peptide-based HIV inactivators. **(A)** Schematic representation of HIV-1 Env composition, including the surface subunit gp120 and the transmembrane subunit gp41. Key residues of CD4bs are located in the region of residues 362–372 in gp120. Amino acid residues are numbered according to those of BG505 SOSIP.664 trimer (PDB ID: 5V8M). **(B)** Attachment of the HIV-1 Env to the cellular receptor(s) and fusion of viral envelope with the target cell membrane. Binding of gp120 with CD4 on the target cell surface triggers conformational change of gp120 with the exposure of the CoRbs, allowing gp120 binding to its coreceptor CCR5 or CXCR4. Subsequently, gp41 changes its conformation, resulting in the insertion of fusion peptide (FP) of gp41 into the target cell membrane and formation of the pre-fusion intermediate (PFI) state. The N- and C-terminal heptad repeats (NHRs and CHRs) of the three gp41 subunits interact with each other to form a six-helix bundle (6-HB) structure, which brings the viral envelope and target cell membrane together to achieve fusion. The pre-fusion trimer is highlighted in a green box, which is shown in more detail in **(C)**. **(C)** Side view of the pre-fusion, trimeric conformation of the Env present on the virion surface, which is presented as the glycan-shielded crystal structure (modified from PDB ID: 5V8M). Names and illustrations of inactivators are drawn in the three boxes, and the arrows depict their target sites, including CD4bs, CD4bs plus CoRbs or PFI induced by D1D2 binding to CD4bs.

Human immunodeficiency virus type 1 (HIV-1) envelope glycoprotein is originally expressed as the gp160 glycoprotein precursor, approximately 850 amino acids in length (Figure 2A), which is cleaved by viral protease to form a highly glycosylated trimer of heterodimers, non-covalently associated by three gp120 and three gp41 subunits (Liu et al., 2008) (Figure 2C). The surface subunit gp120 is approximately 500 amino acids in length, composed of several variable regions V1–V5 and the remaining more conserved regions (Starcich et al., 1986). A conserved binding site to the cellular receptor CD4 (CD4bs) is found on the surface of the gp120 subunit, which contains the conserved hydrophobic Phe-43 pocket (residues 362–372). The transmembrane subunit gp41, approximately 350 amino acids in length, is comprised of the fusion peptide (FP), N-terminal heptad repeat (NHR), C-terminal heptad repeat (CHR), membrane-proximal external region (MPER), transmembrane region (TM), and cytoplasmic region (CP). As shown in Figure 2B, HIV-1 entry is originated by gp120 binding to the CD4 molecule on the target cell, resulting in its conformational change to expose the coreceptor-binding site (CoRbs) on gp120, further allowing gp120 binding to the coreceptor CCR5 or CXCR4 (Chan and Kim, 1998). Subsequently, gp41 also changes conformation by inserting its FP into the target cell membrane to form a prehairpin fusion intermediate conformation (PFI) (Melikyan, 2008). Then, NHRs and CHRs of the three gp41 subunits interact with each other to form a six-helix bundle (6-HB) core structure, bringing the viral envelope and cell membrane into close proximity to achieve fusion (Su et al., 2017). Therefore, the HIV-1 Env composed of gp120 and gp41 subunits is a key component responsible for mediating entry of the virion into the target cell, and also an important target for development of the protein- and peptide-based HIV-1 inactivators (D’Souza et al., 2000).

Lu et al. (2012) proposed, for the first time, the strategy of designing and developing a protein-based HIV-1 inactivator, 2DLT, for inactivating cell-free HIV-1 virions in the absence of target cells (Sanders, 2013). 2DLT, a bivalent recombinant protein, consists of three parts: (1) the D1D2 domain of CD4 (2D), (2) a 35-amino acid linker (L), and (3) T1144 (T), a peptide-based HIV fusion inhibitor (Dwyer et al., 2007; Pan et al., 2009). Binding of D1D2 in 2DLT to the CD4-binding site (CD4bs) in HIV-1 gp120 triggers the formation of a gp120/gp41 pre-fusion intermediate (PFI), in which gp41 NHR is partially exposed (Haim et al., 2009). The T1144 portion in 2DLT then binds the exposed NHR, resulting in the destabilization of PFI thus inactivation of the cell-free virions (Figure 2C). The results from the virus inactivation assay indicated that 2DLT could effectively inactivate cell-free HIV-1 virions, including laboratory-adapted strains and primary isolates of different subtypes with an EC50 (Half maximal effective concentration causing virus inactivation) between 17.3 and 78.6 nM, which is about two–six fold more potent than D1D2 alone, while T1144 alone had no virus inactivation activity (Lu et al., 2012). Therefore, this bivalent protein can actively attack the cell-free virions anywhere in blood when they meet and irreversibly inactivate the cell-free virions through a double hit by targeting gp120 and gp41 simultaneously or sequentially (Lu et al., 2012; Sanders, 2013). Subsequently, the antiviral effects of 2DLT in combination with different anti-HIV drugs were explored, including HIV entry inhibitors, NRTIs, NNRTIs and protease inhibitors. The results indicated that the combination of 2DLT with these drugs brought about synergism or strong synergism against infection of both X4 and R5 HIV-1 strains (Xu et al., 2014).

Actually, several anti-HIV proteins or peptides with HIV inactivation activity similar to that of 2DLT had already been studied before 2011, but without assessing their virus inactivation effects. The first one was soluble CD4 (sCD4) (Deen et al., 1988; Traunecker et al., 1988). Theoretically, sCD4 could interact with the CD4bs in gp120 on HIV-1 and inactivate the cell-free virions in the absence of host cells because virions would lose their ability to bind with the cellular receptor CD4, making it impossible for them to enter the CD4 T cell for replication. Indeed, *in vitro* studies indicated that sCD4 could inhibit HIV-1 infection with IC50 (half maximal inhibitory concentration) values between 40 and 700 nM (Daar et al., 1990; Orloff et al., 1993). The results from phases I–II clinical trials showed that intravenously administered sCD4 effectively reduced viral titers in sera without causing obvious toxicity. However, the half-life of sCD4 is short, leading to viral relapse within a short time after treatment (Kahn et al., 1990; Schooley et al., 1990). At low concentrations (<1 μg/ml or 20 nM), sCD4 could not effectively inhibit HIV-1 infection, but rather enhanced the infection of some HIV-1 clinical isolates in host cells, including some CD4- CCR5+ cells. This is because sCD4 binding to CD4bs in gp120 triggers the exposure of CoRbs in gp120 and NHR in gp41, facilitating the interaction between CoRbs in gp120 and the CCR5 coreceptor on the host cell and, hence, fusion between the viral envelope and host cell membrane (Sullivan et al., 1998). Since CD4 is an important immune molecule that exerts its function through interaction with a number of human proteins, *in vivo* application of sCD4, an anti-HIV drug, may cause some adverse effects to the immune system. Therefore, development of sCD4 for clinical use has been discontinued, but the important information from the research on sCD4 has promoted studies of HIV inactivators containing part(s) of CD4 molecule.

Allaway et al. (1995) constructed a fusion protein CD4-IgG2 comprised of human IgG2 in which the Fv portions of both heavy and light chains were replaced by D1D2 domains of CD4. They found that CD4-IgG2 bound gp120 with high affinity and was much more potent than sCD4 to inhibit HIV-1 fusion and infection. Unlike sCD4, CD4-IgG2 could not enhance HIV-1 infection in CD4- CCR5+ cells. Like the broadly neutralizing antibodies (bnAbs), IgG1b12, 2G12, and 2F5, CD4-IgG2 could effectively neutralize a panel of laboratory-adapted strains and primary isolates of HIV-1 with different subtypes with IC50s from 5 to 80 nM (Trkola et al., 1995), and it was capable of reducing viral titers in rodent models (Gauduin et al., 1998). The results from clinical trials showed that CD4-IgG2 was well-tolerated at doses of 0.2–10 mg/kg with a half-life of 3–4 days *in vivo*, which was much longer than that of sCD4 (Jacobson et al., 2000). In advanced disease, an 80% response rate and ∼0.5 log10 mean reductions in viral load for 4–6 weeks after treatment were mediated by CD4-IgG2 (Jacobson et al., 2004). However, the concern about the enhancement of infection of some HIV-1 strains caused by sCD4- or CD4-containing molecule, like CD4-IgG2, cannot be excluded since no such study has been reported so far.

According to the structural similarity between CD4’s the CDR2-like loop in CD4 and scyllatoxin’s the β-hairpin region of scyllatoxin, a short scorpion toxin, the side chains of nine residues in CD4 that are critical for, central in the gp120 binding to HIV-1 gp120, was transferred to the structurally homologous region in the scorpion toxin scaffold (Vita et al., 1999). The resulting miniprotein (27 residues), CD4M9 inhibited CD4 binding to gp120 with an IC50 of 40 μM and suppressed the infection of HIV-1 X4 and R5 strains with IC50s ranging from 0.4 to 5 μM. Subsequently, they designed bivalent and trivalent CD4-mimetic miniproteins based on CD4M9 and tested their antiviral activity *in vitro*. Results showed that though their antiviral activity was improved, it was still much weaker than that of sCD4 (Li et al., 2004, 2007). Theoretically, these recombinant fusion proteins are expected to possess HIV-1 inactivation activity if a virus inactivation assay is performed.

Since the single D1 domain of CD4 is unstable and has low gp120-binding affinity, Sharma et al. (2005) and Chen et al. (2011) constructed two mutants of the CD4 D1 domain, CD4D1 and CD4PEP1, using mutagenesis. They demonstrated that both CD4D1 and CD4PEP1 could interact with gp120 with KD values of 40 and 26 nM, respectively. However, their virus inhibition or inactivation activities have not been detected and reported. Chen et al. (2011) identified two monomeric D1 mutants with high stability, solubility and gp120-binding affinity, designated mD1.1 and mD1.2, through screening a large D1 mutant library and found that both mD1.1 and mD1.2 could effectively neutralize HIV-1 primary isolates (Sharma et al., 2005). Subsequently, they identified an mD1.2 mutant, mD1.22, which had higher thermostability, better solubility and stronger gp120-binding affinity compared to mD1.2. *In vitro* studies showed that mD1.22 was several folds more potent than D1D2 and mD1.2 in inhibiting R5-tropic HIV-1 primary isolates, Bal and JRFL (Chen et al., 2014). To further improve its anti-HIV-1 activity and breadth, mD1.22 was fused with m36.4, an engineered human antibody domain targeting a CD4-induced (CD4i) epitope, which overlaps the HIV-1 coreceptor-binding site (CoRbs) on gp120 (Chen et al., 2008). This resulted in the generation of two bispecific multivalent fusion proteins, 2Dm2m and 4Dm2m, which could inhibit infection by all HIV-1 strains tested, including 41 HIV-1 isolates circulating predominantly in China, with an average IC50 of 1.7 and 0.2 nM, respectively (Chen et al., 2014). Most recently, the virus inactivation assay was utilized to compare the ability of 2Dm2m and 4Dm2m to inactivate cell-free HIV-1 IIIB virions against full-length sCD4, D1D2 (Sharma et al., 2005; Chen et al., 2011), and mD1.22 (Chen et al., 2014). The results implied that mD1.22, 2Dm2m, and 4Dm2m were highly effective in inactivating HIV-1 with EC50s of 3.1, 1.1, and 0.3 nM, respectively, which are much more potent than those of sCD4 (EC50: 153 nM) and D1D2 (EC50: 64.7 nM) (Qi et al., 2017). These results indicate that the single-domain protein mD1.22 and the bispecific multivalent proteins 2Dm2m and 4Dm2m are important HIV inactivators and are promising to be further developed into new anti-HIV drugs for treatment and prevention of HIV infection.

By screening phage display libraries of random 12-mer peptides, Ferrer and Harrison (1999) and Biorn et al. (2004) identified a 12 amino-acid peptide, 12p1 (RINNIPWSEAMM), which could inhibit gp120 interaction with sCD4 and mAb 17b, a neutralizing antibody that targets the CoRbs on gp120, indicating that 12p1 could simultaneously bind to CD4bs and CoRbs. Later, Ferrer and Harrison (1999) and Biorn et al. (2004) demonstrated that 12p1 could preferentially bind gp120 before gp120engagement of CD4, thus limiting the interaction of gp120 with the receptor CD4 and the coreceptor CCR5, both of which are crucial for viral entry. Therefore, 12p1 may act as an HIV-1 attachment inhibitor and/or inactivator. By optimizing the structure and activity of 12p1, Chaiken and Rashad (2015) designed and synthesized a class of peptide triazoles which could simultaneously bind CD4bs and CoRbs on gp120 with higher affinity than that of 12p1, causing gp120 shedding from the viral particles, and thereby irreversibly inactivating the virions (Aneja et al., 2015). This group has designed and synthesized a new peptide triazole denoted KR13 which can simultaneously induce the shedding of gp120 and the release of capsid protein p24 from HIV-1 pseudoviruses (Bastian et al., 2011, 2013). Later, they found that gold nanoparticle-conjugated KR13 (AuNP-KR13) exhibited more potent virus-inactivating effect than that of peptide KR13 alone because AuNP-KR13 had many more gp120-binding sites than KR13 (Rosemary Bastian et al., 2015).

Most recently, Chaiken and Rashad (2015) designed and engineered a recombinant chimera, denoted DAVEI (dual-acting virucidal entry inhibitor), composed of the lectin cyanovirin-N (CVN) and the HIV-1 gp41 MPER sequence, which could effectively inactivate the HIV-1 pseudovirus Bal.01 with an EC50 value of 28.3 nM. They found that DAVEI exhibited potent and irreversible inactivation of HIV-1 virions by dual engagement of gp120 and gp41, while CVN or MPER alone had no HIV-1 inactivation activity (Parajuli et al., 2016, 2018). These studies provided rational approaches for the design and development of specific HIV-1 inactivators with improved antiviral activity for treatment and prevention of HIV-1 infection.

The CD4bs on gp120 appear to be the most important target of protein- and peptide-based HIV inactivators. Most of those that are under preclinical and clinical development target CD4bs alone, CD4bs plus co-receptor-binding site (CoRbs) on gp120, or CD4bs plus some region(s) in the HIV-1 gp41 (Figure 2C). These strategies can also be applied to design and develop virus inactivators against other enveloped viruses, such as those described below.

### Protein- and Peptide-Based Influenza Virus Inactivators

Influenza viruses belong to the *Orthomyxoviridae* family having segmented negative-sense, single-stranded RNA genomes (Palese and Shaw, 2007). Influenza virus infection can lead to a high fever, cough, headache, sore throat etc. after 1–3-day incubation period. Influenza viruses are classified as types A, B, C, and D, on the basis of antigenic properties of the viral nucleoprotein (NP) and matrix protein (M). Since most annual influenza epidemics in humans are caused by influenza A viruses (IAVs) (Dou et al., 2018), we mainly discuss IAVs here. A vital challenge in combating IAVs is the constant evolution of the surface antigens, hemagglutinin (HA) and neuraminidase (NA), to resist pressure from the host immune system, which is described as antigenic drift and antigenic shift. Therefore, IAVs are classified into subtypes based on the genetic and antigenic differences of HA and NA, including 18 subtypes of HA (H1–H18) and 11 subtypes of NA (N1–N11) (Gamblin and Skehel, 2010).

At present, there are mainly two types of anti-influenza drugs used in clinics, including (1) M2 ion channel inhibitors, such as amantadine (Bright et al., 2006) and rimantadine, which block viral uncoating, and (2) neuraminidase inhibitors (NAIs), including oseltamivir (de Jong et al., 2005), zanamivir, peramivir, and laninamivir octanoate, which inhibit viral release. However, the continual emergence of drug resistance seriously limits their effectiveness and clinical applications (Zeng et al., 2017). In 2018, a new antiviral drug, baloxavir marboxil (trade name Xofluza), which can inhibit viral replication in cells, was approved by Japanese and United States FDA and proved effective against infection by influenza virus strains resistant to current anti-influenza drugs (Ng, 2019). Still, several cases of drug resistance to baloxavir marboxil have been reported recently (Takashita et al., 2019; Sato et al., 2020). Also, these drugs mentioned above must inhibit viral infection in the presence of target cells, instead of inactivating cell-free influenza virions.

**FIGURE 3:**
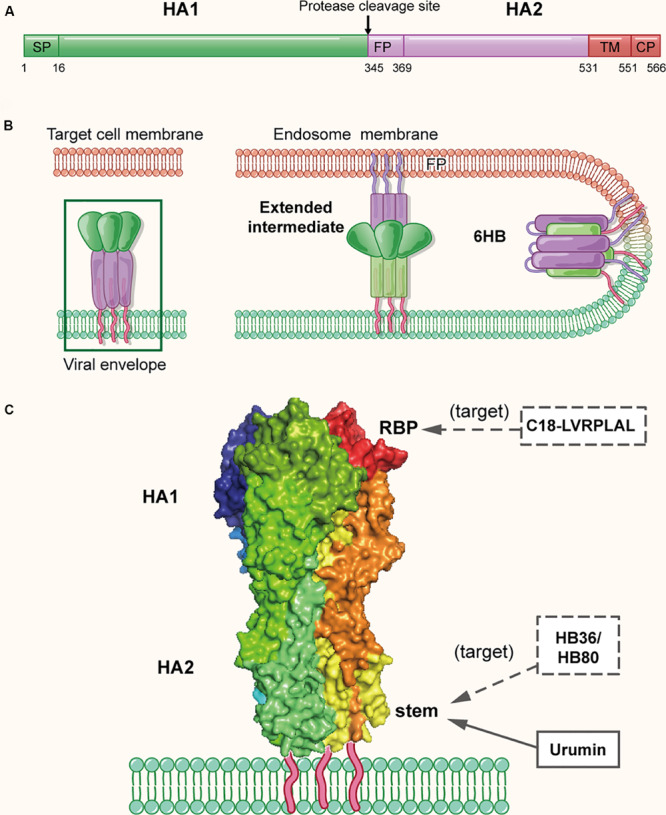
The structure of influenza virus HA protein in the native and fusion-intermediate states, which serve as the target of the protein- and peptide-based influenza virus inactivators. **(A)** Schematic representation of HA composition, including surface subunit HA1 and transmembrane subunit HA2. **(B)** Attachment of HA protein to the cellular receptor(s) and fusion of viral envelope with endosomal membrane. To initiate the entry process, the RBS on HA attaches the virus to cell surface receptors that contain terminal sialic-acid residues, followed by entry of the virion into an endosome of the host cell via endocytosis. The acidic environment in the endosome induces conformational change of HA, enabling the exposure of the fusion peptide on the N-terminus of HA2. Subsequently, the fusion peptide inserts into the endosomal membrane with the C-terminal transmembrane domain (TMD) anchoring HA2 in the viral envelope, creating a pre-hairpin conformation (extended intermediate). Afterward, the HA2 trimers fold back on themselves forming a hairpin conformation, which then further collapses into a six-helix bundle, bringing the viral envelope and endosomal membrane together for fusion. The pre-fusion trimer is highlighted in a green box, which is shown in more detail in **(C)**. **(C)** Side view of the pre-fusion, trimeric conformation of HA protein present on the virion surface (modified from PDB ID: 4FNK). The heads of HA1 subunits are colored with dark tints, showing the receptor-binding pockets (RBPs). Names of inactivators are listed in the three boxes, and the arrows depict their target sites, including the conserved stem and RBP. Notably, potential inactivators remaining to be tested are listed in the box with dashed lines and arrow.

As mentioned above, two envelope glycoproteins, HA and NA, are on the surface of the influenza virion. HA is originally translated as the HA0 precursor, about 560 amino acids (Figure 3A). Subsequently, it undergoes proteolytic cleavage and glycosylation to form a heterodimerized trimer composed of three HA1 and three HA2 subunits, and approximately 350–400 trimers are found on the surface of a virion (Skehel and Wiley, 2000). On the surface of HA1 globular head, it is the RBS (residues 116–261), composed of a β-barrel motif and α-helices, which resembles a shallow pocket. The key receptor binding residues are highly conserved among different HA subtypes (Priyadarzini et al., 2012). The C-terminus of HA2 subunit is the conserved stem, which is the target of multiple neutralizing antibodies (Ekiert et al., 2009; Sui et al., 2009). As shown in Figure 3B, to initiate the entry process, the RBS on HA attaches the virus to cell surface receptors that contain terminal sialic-acid residues, which triggers the virion entering an endosome via endocytosis (Sun and Whittaker, 2013). The acidic environment in the endosome induces conformational change of HA, enabling the exposure of the fusion peptide on the N-terminus of HA2. Subsequently, the fusion peptide inserts into the endosomal membrane with the C-terminal transmembrane domain (TMD) anchoring HA2 in the viral envelope, to create a pre-hairpin conformation (Bullough et al., 1994). Afterward, the HA2 subunit fold back on itself to form a hairpin that brings the two membranes closer. The hairpin further collapses into a six-helix bundle, which enables the formation of the lipid stalk and the subsequent fusion of the two membranes (Harrison, 2008, 2015).

Because HA plays a key role in viral attachment and membrane fusion, it is the promising target for the development of protein- and peptide-based IAV inactivators. Recently, Holthausen et al. (2017) isolated an amphibian special host defense peptide (HDP), urumin, from skin secretions of a frog native to southern India. This is a 27-amino acid peptide with net positive charges. Different from most HDPs that exert their virucidal activity by non-specific interaction with the viral lipid membranes, urumin could specifically interact with the conserved stalk region of H1N1 HA and then destroy the influenza virions. Interestingly, *in vivo* application of urumin could protect naive mice from challenge with a lethal dose of IAV infection, suggesting that urumin has good potential for further development into a first-line antiviral treatment during influenza outbreaks.

Using a computer-aided strategy based on the structural information of the binding site of neutralizing antibodies (Ekiert et al., 2009; Sui et al., 2009), Baker and colleagues designed proteins named HB36 and HB80, which could bind to a conserved surface patch of the HA stem from the 1918 H1N1 pandemic virus (Fleishman et al., 2011). Results showed HB36 and HB80 bound H1 and H5 HAs with low nanomolar (nM) affinity. Based on their mechanism of action, proteins designed in this way are expected to possess viral inactivation abilities; however, a viral inactivation experiment was not performed in this study. Therefore, the actual inactivation activities of HB36 and HB80 were uncertain, which requires further investigation.

In addition to the conserved stem of HA, the receptor-binding pocket mentioned above is also a promising target of IAV inactivators (Whittle et al., 2011; Ekiert et al., 2012; Yang et al., 2013) (Figure 3C). Through screening a phage-displayed random peptide library, Matsubara et al. (2010) identified several 15-mer sialic acid-mimic peptides that could bind the RBSs in H1 and H3 HAs and inhibit infection by the A/Puerto Rico/8/34 (H1N1) and A/Aichi/2/68 (H3N2) strains of IAV with IC50 at low μM level. Later, they identified a series of 7-mer sialic acid-mimic peptides (Matsubara et al., 2016) by screening another phage-displayed random peptide library. They found that these peptides could also bind H1 and H3 HAs and that the binding could be inhibited in the presence of sialic acid. Plaque assays indicated that one of these peptides, C18-LVRPLAL, could strongly inhibit infection by the A/Aichi/2/68 (H3N2) strain with an IC50 value of 6.4 μM. However, none of the above peptides have been tested for their virus inactivation activities using a virus inactivation assay.

In sum, two conserved sites on HA protein, including the receptor-binding pocket and the conserved stem, have been the promising targets of influenza virus inactivators. Although HA protein itself does not belong to the range of inactivators we discuss here for not acting on cell-free virions, it can function through other two ways. It can be the possible receptor antagonist, which binds to the cell receptor to inhibit viral infection. Also, it has been the key immunogen of currently licensed influenza vaccines (Bosch et al., 2010; Houser and Subbarao, 2015; Yamada et al., 2019), which could elicit strain-specific anti-HA antibodies to neutralize the virus and prevent or control infection. Moreover, since most agents mentioned above have not been testified to possess inactivation activities against influenza virus with a standard assay, there still remains a long way to put them into practice.

### Protein- and Peptide-Based ZIKV and DENV Inactivators

Flaviviruses, with a positive-sense single-stranded RNA genome, are enveloped viruses transmitted by hematophagous mosquito vectors. Flavivirus infection may cause neurological, viscerotrophic or hemorrhagic diseases (Slon Campos et al., 2018). The *Flavivirus* genus (Flaviviridae family) is comprised of a variety of human pathogens, including DENV, ZIKV, yellow fever virus (YFV), West Nile virus (WNV), and so on (Kuno et al., 1998).

Zika virus infection usually causes mild symptoms, such as rash, fever, headache, and joint pain, but severe symptoms in some cases, such as Guillain-Barré syndrome, meningo-encephalitis and myelitis. ZIKV infection of pregnant women may cause microcephaly in their fetuses and newborns (Cui et al., 2017; Culshaw et al., 2018). Currently, with repurposing approaches, several FDA-approved drugs with anti-ZIKV activities have been identified, such as Sofosbuvir (Xu et al., 2016), but no drug has been licensed for clinical use (Wang et al., 2017; Han and Mesplede, 2018). Four serotypes of DENV (DENV-1, 2, 3, and 4) constitute the primary mosquito-borne viral pathogen. DENV is endemic in more than 100 countries worldwide, and infects ∼390 million people each year, of which 96 million people exhibit disease symptoms (Bhatt et al., 2013). After DENV infection, some individuals exhibit mild symptoms like flu, while others might suffer from more severe diseases, such as dengue hemorrhagic fever and shock syndrome (DHF/DSS) (Halstead, 2007). Till now, only several small molecule anti-DENV drugs such as UV-4B (Warfield et al., 2016) have entered Phase I or Phase II clinical trials (Tian et al., 2018), and no specific anti-DENV drugs have been approved for clinical use yet.

**FIGURE 4:**
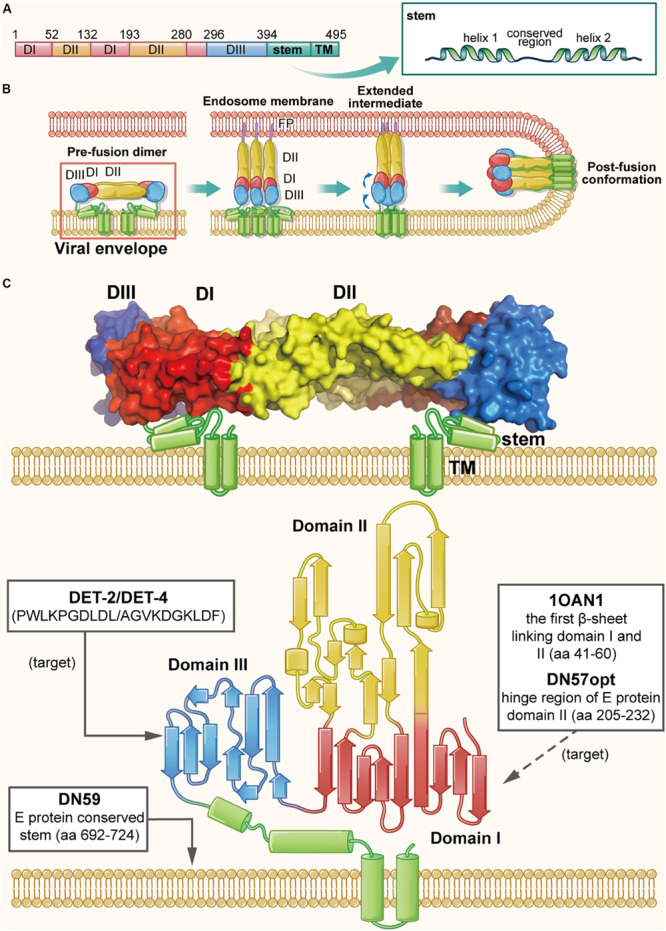
The structure of DENV E protein in the native and fusion-intermediate states, which serve as sources and targets of the protein- and peptide-based DENV inactivators. **(A)** Schematic representation of E protein composition, including domains I–III (colored red, yellow and blue, respectively), stem and transmembrane region (TM). **(B)** Attachment of DENV E protein to the cellular receptor(s) and fusion of viral envelope with the endosome membrane. Binding of E protein domain III with the receptor on the target cell is followed by entry of the virion into endosomes of the host cell via endocytosis. Under the acidic environment in the endosome, E protein dimer dissociates, resulting in the individual subunits swinging outward. The exposed fusion loops insert into the endosomal membrane, which facilitates the reassembling of the subunits to form the extended, trimeric intermediate. Subsequently, domain III in each subunit is reversely folded to promote the viral envelope and the endosomal membrane into close proximity for fusion. The pre-fusion dimer is highlighted in a red box, which is shown in more detail in **(C)**. **(C)** Side view of the pre-fusion, dimeric conformation of E protein present on the virion surface (modified from PDB ID: 1OAN). Names and sequences of inactivators are listed in the three boxes, and the arrows depict their target sites, including the lateral loop of domain III and the viral lipid membrane. Notably, potential inactivators remaining to be tested are listed in the box with a dashed arrow.

The particle structure and genomic organization are similar among all flaviviruses, with 180 E proteins and 180 M proteins forming 90 heterodimers, covering most surface area of a virion (Slon Campos et al., 2018). Taking DENV as an example, the ectodomain of each E protein monomer consists of three domains I, II, and III. Domain II contains a highly hydrophobic fusion loop capable of mediating fusion, and domain III contains RBSs. The C-terminus of domain III is the stem, a membrane anchoring region (Figure 4A), which is highly conserved in flaviviruses and forms a helix-loop-helix structure located below the E protein ectodomain and partially embedded in the viral envelope (Figure 4C) (Zhang et al., 2013). The M protein, consisting of 75 amino acids, includes a 40-amino acid ectodomain and a 35-amino acid transmembrane region. The ectodomain includes a hydrophobic loop structure, followed by an amphipathic helix, which may interact with the E protein on the virion surface (Zhang et al., 2013). Analysis of the interaction between E and M proteins on the DENV surface revealed that M protein, functioning as a pH-sensitive chaperone, plays a vital role during the process of viral infection and maturation (Li et al., 2008). As shown in Figure 4B, DENV infection is initiated by binding of domain III of E protein to the receptors on the target cell (Huerta et al., 2008), followed by entry of the virion into the endosome in the host cell via endocytosis. Under the acidic environment in the endosome, the E protein dimer dissociates, resulting in the individual subunits swinging outward. The exposed fusion loops insert into the endosomal membrane, promoting reassembly of the subunits to form the extended, trimeric intermediate. Subsequently, domain III in each subunit is reversely folded, bringing the viral envelope and the endosomal membrane into close proximity for fusion (Harrison, 2008; Klein et al., 2013).

Since E protein plays an significant role in receptor binding and membrane fusion, it is considered as a promising source and target for development of protein- and peptide-based DENV inactivators. In recent years, the structure of DENV E protein has been well determined using X-ray crystallography and cryo-electron microscopy (Cryo-EM) (Zhang et al., 2013), which has allowed researchers to rationally design DENV inactivators (Chew et al., 2017). Using a physiochemical algorithm, the Wimley-White interfacial hydrophobicity scale (WWIHS), together with the known structural information of DENV E protein, Hrobowski et al. (2005) designed four peptides (DN80, DN57, DN81, and DN59) derived from different regions of DENV E protein. They found that DN59, derived from the helix-loop-helix sequence of the DENV-2 E protein stem region (residues 692–724) with amphipathicity and membrane-binding ability, could inhibit infection of all four serotypes of DENV with IC50 values in the range of 2–5 μM. It was also effective against some other flaviviruses, such as yellow fever virus (YFV) (Lok et al., 2012). Further studies of its mechanism of action have shown that it can irreversibly inactivate virions by directly interacting with viral lipid membranes and forming holes at the five-fold vertices in the viral envelope, allowing the release of viral genomic RNA (Schmidt et al., 2010a, b; Lok et al., 2012).

Since the stem of flavivirus E protein is highly conserved, results of the above studies imply that protein- and peptide-based ZIKV inactivators can also originate from the stem sequence. Accordingly, peptide Z2 was designed and synthesized, which is derived from the stem of ZIKV E protein (residues 421–453), and similarly Z2 was shown to inhibit infection by different strains of ZIKV and other flaviviruses, including YFV and DENV with IC50 values between 1 and 14 μM. Animal studies indicated that Z2 could protect A129 and AG6 mice from lethal ZIKV challenge; Z2 was also found to cross the placental barrier and prohibit vertical transmission of ZIKV in pregnant mice. The mechanism studies suggested that Z2 could interact with E protein and inactivate the virions, with an EC50 value of ∼2.5 μM, by forming pores in the viral envelope, allowing the release of the viral RNA genome (Yu et al., 2017). Although Z2 could not cross the blood–brain barrier (BBB) to enter the fetal brain, it was able to inactivate virions in the placenta or umbilical cord, thus effectively reducing the viral titers in the fetal brain.

In addition to the conserved stem, other regions in E protein may also serve as sources of DENV inactivators. Based on the native dimeric E structure, Costin et al. (2010) designed a set of anti-DENV peptides using predictive strategies together with computational optimization. The two most active peptides, DN57opt and 1OAN1, derived from the domain II hinge region (residues 205–232) and the first domain I/domain II beta sheet connection (residues 41–60), respectively, could inhibit DENV-2 infection with IC50 values of 8 and 7 μM, respectively. The biolayer interferometry study demonstrated that both DN57opt and 1OAN1 could bind to soluble DENV-2 E protein, and Cryo-EM analysis revealed that the surface of virions treated with DN57opt or 1OAN1 became rough, suggesting that the viral envelope may have been damaged (Costin et al., 2010). However, the virus inactivation abilities of these peptides were not reported, and their specific mechanism of action needs to be further explored.

Apart from being the source of DENV inactivators, other regions of E protein can also be potential targets. A previous study reported the existence of a hydrophobic pocket between domain I and II, which acted as a hinge of the E protein structural rearrangement and could interact with the detergent β-*N*-octyl-glucoside (Modis et al., 2003). By molecular docking analysis, Panya et al. (2014) identified several short peptides targeting the hydrophobic pocket. They found that the dipeptide Glu-Phe (EF for short) could inhibit the infection of all four DENV serotypes, but it was the most effective against DENV-2 with an IC50 value of 96 μM. Different inhibitory effect for the four serotypes of DENV was possible because of the different amino acid sequence of their hydrophobic pockets. Inferred from its mechanism of action, EF peptide can be considered as a DENV inactivator, as potentially confirmed by performing a virus inactivation assay. In addition, the lateral loop of domain III has been verified to play an important role in virus-cell receptor interaction, thus making it another feasible target of protein- and peptide-based DENV inactivators (Hung et al., 2004; Mazumder et al., 2007). Using the BioMoDroid algorithm, Alhoot et al. (2013) screened for anti-DENV peptides targeting a short sequence (residues 380–389) in the lateral loop of DENV-2 E protein domain III. They found that peptides DET2 and DET4 could inhibit DENV-2 infection with IC50 values of 500 and 35 μM, respectively. Observed with transmission electron microscopy, the surface of virions was found to become irregular and have rough edges, suggesting that these peptides could inactivate virions by disrupting integrity of the viral envelope.

Other studies showed that M protein of DENV could interact with native E protein to trigger the conformation of DENV E protein, suggesting that peptides derived from M protein may act as DENV inactivators. Panya et al. (2015) designed a novel anti-DENV peptide, MLH40, derived from the conserved ectodomain of M protein, and found that it could inhibit infection of four serotypes of DENV with IC50 values ranging from 24 to 31 μM. Molecular docking analysis suggested that the N-terminal loop of MLH40 could interact with DENV E protein to alter its native dimeric conformation. However, its inactivation activity has not been tested and reported.

In sum, the studies on DENV and ZIKV inactivators are not as comprehensive as those on HIV inactivators (Figure 4C). Almost all of them are peptides, not proteins; some important targets including the RBS in domain III have not been utilized adequately like CD4bs in HIV gp120 (Huerta et al., 2008). In addition, the viral inactivation activities of many peptides have not been verified with the virus inactivation assay, and specific targets and action mechanisms of most peptide-based inactivators have not been clearly elucidated. Despite of these deficiencies, the design and development of DENV and ZIKV inactivators can be referential paradigms for those of newly emerging or re-emerging viruses.

### Protein- and Peptide-Based HSV Inactivators

Herpesviruses, a large and diverse family of enveloped viruses with double-stranded DNA genomes, can bring about lifelong, latent infections (Pellet and Roizman, 2013). These viruses are classified as three subfamilies, alpha-, beta-, and gamma-, on the basis of their genome sequences and biological properties (Davison et al., 2009). In alpha-herpesviruses, herpes simplex virus types 1 and 2 (HSV-1 and HSV-2) and varicella-zoster virus (VZV) are human pathogens, which routinely infect humans (Roizman et al., 2013). HSV, among the most widespread pathogenic agents in the human population, causes varieties of diseases, ranging from oral and genital ulcers to devastating encephalitis. Following the initial infection at a peripheral site, it will establish a lifetime latency in sensory neurons, which can be reactivated by some internal or external stimuli, including fever, immunosuppression, trauma, etc. (Roizman et al., 2013). So far, three categories of drugs have got approval for the treatment of HSV infection, including (1) the nucleoside analogs such as acyclovir (ACV), (2) the acyclic nucleotide analog cidofovir and (3) the pyrophosphate analog foscarnet (Kimberlin and Whitley, 2007). Till now, ACV remains the prototypic antiviral agent and the reference for treating HSV infection. However, viral resistance to ACV has become a critical clinical problem, especially concerning immunocompromised patients undergoing long-term therapy (Englund et al., 1990). Although the other two drugs also have been proven to be effective for treating HSV infection, they are reserved for confirmed cases of ACV resistance due to their nephrotoxicity (Galdiero et al., 2013). In addition, these drugs all exert antiviral effects by targeting viral DNA polymerase in target cells, therefore no antiviral drugs targeting HSV entry are available currently, let alone inactivating virions (Galdiero et al., 2013).

**FIGURE 5:**
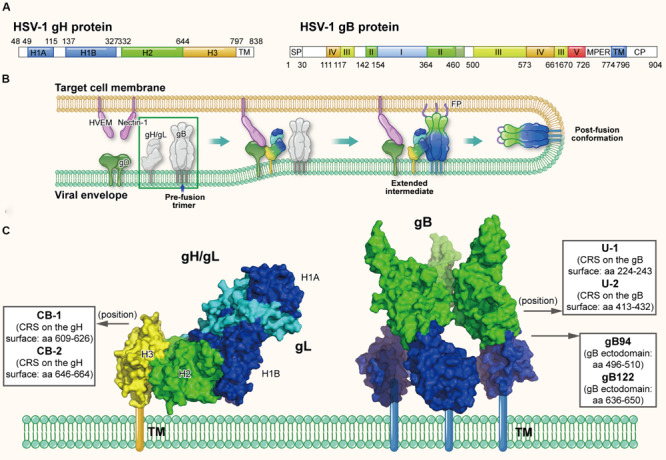
The structures of HSV gH and gB protein in the native and fusion-intermediate states, which serve as sources of protein- and peptide-based HSV inactivators. **(A)** Schematic representation of HSV-1 gH and gB composition. gH is composed of the ectodomain (H1, H2, and H3 domains, colored mazarine blue, green, and yellow, respectively), transmembrane domain (TM) and cytoplasmic tail (CP). gB is composed of domains I–V, transmembrane region (TM) and cytoplasmic domain (CP). **(B)** The HSV entry process orchestrated by a set of glycoproteins, including receptor-binding gD, fusion-regulating gH/gL and the fusogen gB. gD binding to either receptor, such as HVEM and nectin-1, causes its conformational change to expose the profusion domain. gB may also bind to specific cell receptors, such as (PILR)α and NMHC-II. Meanwhile, gH/gL may be facilitated by the activated form of gD to convert into a form able to interact with gB, triggering the transformation of the prefusion form of gB into a fusogenic state. Next, Akt (protein kinase B) may be triggered to translocate to the outer leaflet microdomains of the plasma membrane to interact with gB, which further induces Akt phosphorylation and intracellular calcium release. Subsequently gB inserts the fusion loop into the target cell membrane to form the extended, trimeric intermediate, which allows fusion of the two membranes. The pre-fusion conformation of gH/gL and gB are highlighted in a green box, which is shown in more detail in **(C)**. **(C)** Side view of the pre-fusion conformation of the native gH/gL complex (modified from PDB ID: 3M1C) and gB (modified from PDB ID: 5FZ2). Names and sequences of inactivators are listed in the three boxes, and the arrows depict their positions on gH/gL and gB. Target sites of these inactivators have not been defined clearly, thus not shown here.

Different from most of other enveloped viruses using a single Env to mediate viral fusion process, HSV utilize a set of surface glycoproteins including gD, gH/gL, and gB as the core fusion components. Therein gD acts as the receptor-binding protein, gH/gL as the fusion regulator and gB as the class III fusogen (Heldwein et al., 2006; Arii and Kawaguchi, 2018). Taking HSV-1 as the example, HSV-1 gD (369-amino acids) is a type I membrane glycoprotein with a short cytoplasmic tail and an ectodomain with an immunoglobulin-like core flanked by N- and C-terminal extensions (Carfi et al., 2001). As shown in Figure 5A, HSV-1 gH is an 838-amino acid type I membrane glycoprotein comprised of a single pass transmembrane domain, a short cytoplasmic tail and an ectodomain (Gompels and Minson, 1986). Its ectodomain can be subdivided into domains H1 (H1A and H1B), H2 and H3, and the N-terminal H1 domain binds gL to form a non-covalent heterodimer (Gompels and Minson, 1986; Chowdary et al., 2010). Results showed the crystal structure of gH/gL complex does not resemble any other known viral fusogen structure. Therefore, the gB-gH/gL complex is key for fusion and can be inhibited by neutralizing antibody to gH, implying that the gH/gL complex activates gB through direct binding (Chowdary et al., 2010). Furthermore, a recent study confirmed the C-terminal domain H3 of HSV-1 is important for interaction with gB (Bohm et al., 2016). HSV-1 gB, is a 904-amino acid glycoprotein with an extended rodlike ectodomain, transmembrane domain (TM) and cytoplasmic domain (CP) (Figure 5A). The ectodomain has five distinct parts, domains I–V, and domain IV is fully exposed with neutralizing epitopes on it and possibly interacts with cellular receptors (Heldwein et al., 2006).

Herpes simplex virus may enter cells through endocytosis or direct fusion of the viral envelope with cellular membrane, relying on the target cell type (Nicola, 2016); notably, both routes require the same set of viral glycoproteins (Nicola and Straus, 2004). HSV entry through direct fusion is a coordinated process that involves a cascade of events, which can be briefly divided into (1) binding to specific cell receptors, (2) intracellular signaling, and (3) fusion of the viral envelope with host cell membrane (Connolly et al., 2011). At the beginning of entry process, binding of glycoprotein D to one of its cognate receptors is significant. Four gD receptors of HSV-1 and HSV-2 have been confirmed: a member of the tumor necrosis factor (TNF) receptor family, herpesvirus entry mediator (HVEM); the poliovirus receptor family, including nectin-1 and nectin-2, belonging to the immunoglobulin superfamily; and a modified form of heparan sulfate, 3-*O*-sulfated heparan sulfate, 3-*O*-HS (Weed and Nicola, 2017). Binding of gD to either receptor results in its C-terminus movement, which exposes the profusion domain of gD (residues 260–285) (Cocchi et al., 2004; Krummenacher et al., 2005; Gallagher et al., 2013). Recent studies demonstrated gB may also bind to specific cell receptors, such as paired immunoglobulin-like type 2 receptor (PILR)α (Satoh et al., 2008), myelin-associated glycoprotein (MAG) (Suenaga et al., 2010), non-muscle myosin heavy chain IIA (NMHC-IIA) (Arii et al., 2010) or non-muscle myosin heavy chain IIB (NMHC-IIB) (Arii et al., 2015). Meanwhile, gH/gL may be facilitated by the activated form of gD to convert into a conformation able to interact with gB (Atanasiu et al., 2007, 2010a,b, 2016), triggering the prefusion form of gB transforming into a fusogenic state. Next, Akt (protein kinase B) may be triggered to translocate to the outer leaflet microdomains of the plasma membrane to interact with gB (Cheshenko et al., 2013, 2018), which further induces Akt phosphorylation and intracellular calcium release. Subsequently gB inserts the fusion loop into the target cell membrane (Zeev-Ben-Mordehai et al., 2016) to form the extended, trimeric intermediate, which results in fusion of the two membranes and release of the capsid into the cell (Figure 5B) (Eisenberg et al., 2012; Arii and Kawaguchi, 2018).

Since the direct fusion is governed by gD, gH/gL, and gB, they are potential sources and targets of protein- and peptide-based HSV inactivators (Galdiero et al., 2013). Akkarawongsa et al. (2009) identified three antiviral peptides, gB94, gB122, and gB131, from the peptide library of HIV-1 gB-1 ectodomain (Figure 5C). The peptides gB94 (residues 496–510) and gB122 (residues 636–650) could inactivate cell-free virions with EC50 of 125 and 138 μM, respectively, although their detailed mechanism of action has not been reported. However, while peptide gB131 (residues 681–695) exhibited no virus inactivation, it did show inhibitory activity (IC50: ∼12 μM). More recently, Cetina-Corona et al. (2016) created a library of continuous 15-25 residue stretches (CRSs) located on the surface of HSV-2 gH and HSV-1 gB through bioinformatics analysis. According to the flexibility, charged residues and conservatism of the CRS sequence, they selected and synthesized peptides CB-1 and CB-2 derived from gH and peptides U-1 and U-2 derived from gB (Figure 5C). Results showed that peptides U-1 and U-2 at 100 μM could inactivate more than 80% of HSV-1 and HSV-2 virions, while peptides CB-1 and CB-2 at 100 μM could only inactivate less than 50% of these virions. However, the detailed mechanism of action showing how these peptides inactivate cell-free HSV virions has not been clarified. Meanwhile, the viral inactivation activities of these peptide inactivators are relatively weak, requiring further optimization.

As mentioned above, the fusion process is mediated by a set of interactions between gD, gH/gL, and gB, and between these glycoproteins and their receptors (Azab and Osterrieder, 2017; Weed and Nicola, 2017), thus multiple glycoprotein or RBSs are potential to be targets of HSV inactivators. Taking the potent gH/gL-specific neutralizing antibody as the example, it could inhibit the formation of the gB-gH/gL complex, suggesting that the gB binding site in gH/gL may locate in the vicinity of the neutralizing epitope (Chowdary et al., 2010; Bohm et al., 2016). Therefore, the neutralizing epitope in gH/gL may serve as one of the targets. Similarly, glycoprotein-binding sites or RBSs on gD (Di Giovine et al., 2011; Lee et al., 2013; Cairns et al., 2019) also prompt the design of HSV inactivators, just like the CD4bs in HIV gp120. Therefore, more comprehensive studies on the crystal structures of these glycoproteins and the mechanism of the fusion process are essential for the design of protein- and peptide-based HSV inactivators.

## Discussion

The lower eukaryotic organisms, such as invertebrates and plants, only possess the innate immunity system, including some antimicrobial peptides (AMPs) that can non-specifically destroy the viral envelope and inactivate the cell-free virions (Ganz, 2003; Seo et al., 2012). After long evolution, higher vertebrates have gained an advanced adaptive immune system that can specifically attack and inactivate pathogenic viruses. Neutralizing antibodies are a vital component of the adaptive immune system. According to our definition of virus inactivators described before, many of the viral neutralizing antibodies with the ability to irreversibly inactivate cell-free virions can be considered as protein-based virus inactivators. The development of neutralizing antibodies during the evolutionary process may also reflect the fact that protein-based virus inactivators able to actively attack and inactivate cell-free virions have certain advantages against viral infections (Burton, 2002). Nonetheless, how these neutralizing antibodies inactivate cell-free virions is still unclear, and for this reason, they were not discussed in detail in this review.

Viral fusion proteins in different oligomeric states and structures can be classified into three different classes (classes I, II, and III) (Weissenhorn et al., 2007; Harrison, 2008). Here we reviewed the development of PPVIs against the following representative enveloped viruses: HIV and influenza virus (with class I membrane fusion), ZIKV and DENV (with class II membrane fusion), and HSV (with class III membrane fusion). Although their fusion/entry processes vary from each other, some common technologies can be used for identifying or designing and evaluating PPVIs, including those (1) for constructing recombinant fusion proteins, such as those applied to design the potent protein-based HIV-1 inactivators 2DLT and 2Dm2m (Lu et al., 2012; Chen et al., 2014); (2) for constructing phage display peptide libraries, such as the one utilized to screen for the peptide 12p1, from which the peptide-based HIV-1 inactivator Triazole KR13 was designed (Bastian et al., 2011, 2013); (3) for performing computer-aided design of proteins or peptides targeting the RBS, such as the CD4bs in HIV-1 gp120 and sialic-acid binding site in influenza virus HA1, or the conserved stem regions, such as those in DENV and ZIKV E protein; and (4) for performing viral inactivation assays, such as those for evaluating inactivation activities of the PPVIs against HIV-1 and ZIKV (Lu et al., 2012; Yu et al., 2017). In general, these techniques and strategies have been successfully applied to design a variety of virus inactivators targeting viral Envs, and they can also be used for the design of virus inactivators against other enveloped viruses, particularly the newly emerging and re-emerging viruses with potential to cause global pandemics (Table 1).

**TABLE 1:** Summary of protein- and peptide-based virus inactivators.

Name	Sequence	Target	EC50* (virus tested)	Reference
**HIV inactivators**				
sCD4	Extracellular D1–D4 or D1D2 domain	gp120 CD4bs	153 and 297 nM (HIV-1 IIIB and Bal, respectively)	Deen et al., 1988; Traunecker et al., 1988; Daar et al., 1990; Orloff et al., 1993
2DLT	D1D2 domain-L35-T1144	gp120 CD4bs + gp41 PFI	17.3–78.6 nM (multiple HIV-1 strains)	Lu et al., 2012
2Dm2m/4Dm2m	2 or 4 mD1.22 with 2 m36.4	gp120 CD4bs + CoRbs	0.3–1.1 nM (HIV-1 IIIB)	Chen et al., 2014; Qi et al., 2017
Triazole KR13	Modified peptide derived from 12p1 (RINNIPWSEAMM)	gp120 CD4bs + CoRbs	0.5–25.6 μM (multiple HIV-1 strains)	Bastian et al., 2011, 2013
DAVEI	CVN + gp41 MPER	gp120 glycan sites + gp41	28.3 nM (HIV-1 Bal.01 pseudovirus)	Parajuli et al., 2018
**Influenza virus inactivators**				
Urumin	IPLRGAFINGRWDSQCHRFSNGAIACA	H1 HA conserved stem	HNBD^#^	Holthausen et al., 2017
**ZIKV inactivators**				
DN59	E protein stem (aa 692–724): MAILDDTAWDFGSLGGVFTSIGKALHQ VFGAIY	DENV lipid membrane	4.8 μM (DENV-2)	Schmidt et al., 2010a, b; Lok et al., 2012
Z2	E protein stem (aa 421–453): MAVLGDTAWDFGSVGGALNSLGKGIH QIFGAAF	ZIKV lipid membrane	2.52 μM	Yu et al., 2017
**HSV inactivators**				
gB94	gB ectodomain (aa 496–510): KTTSSIEFARLQFTY	HSV-1	125 μM	Akkarawongsa et al., 2009
gB122	gB ectodomain (aa 636–650): GHRRYFTFGGGYVYF	HSV-1 and 2	118 μM	Akkarawongsa et al., 2009
U-1	CRS on gB surface (aa 224–243): HRDDHETDMELKPANAATRT	HSV-1 and 2	HNBD^#^	Cetina-Corona et al., 2016
U-2	CRS on gB surface (aa 413–432): CIGKDARDAMDRIFARRYNA	HSV-1 and 2	HNBD^#^	Cetina-Corona et al., 2016
CB-1	CRS on gH surface (aa 609–626): QATRSETPVEVLAQQTHG	HSV-1 and 2	HNBD^#^	Cetina-Corona et al., 2016
CB-2	CRS on gH surface (aa 646–664): PEASHRCGGQSANVEPRIL	HSV-1 and 2	HNBD^#^	Cetina-Corona et al., 2016)

*EC50, half maximal effective concentration causing virus inactivation. ^#^HNBD, has not been defined. CD4bs, CD4-binding site; CoRbs, coreceptor-binding site.

Moreover, by learning from the design strategies of inactivators against enveloped viruses, we can now design and develop viral inactivators targeting key proteins involved in the entry process of non-enveloped viruses. The entry process of a non-enveloped virion is initiated by binding of the viral capsid protein to the cell receptor, followed by entry of the virion into endosomes in the host cell through clathrin- or caveolae-mediated endocytosis or macropinocytosis. The low pH environment causes conformational change of the virus capsid, leading to the externalization of membrane-penetrating peptides (MPPs) in endosomal compartments. Finally, MPPs are integrated or associated with endosomal membranes, leading to distortion and disruption of the membrane and allowing the release of nucleocapsid or genome into the cytosol (Kumar et al., 2018). Considering that most complicated fusion steps occur in the cell endosome, the receptor-binding domains in the viral capsid protein seem to be the best targets for design of PPVIs against non-enveloped viruses. However, the structure and function of the receptor-binding domains in their capsid proteins have not been well studied (Day and Schelhaas, 2014). Therefore, the design of virus inactivators against non-enveloped viruses still has a long way to go.

At present, development of PPVIs has become a topic of strong interest in the field of antiviral drugs. Different from “passive defenders,” and fusion inhibitors and receptor antagonists in “gate keepers,” protein- and peptide-based inactivators have been found to actively attack and inactivate cell-free virions anywhere they meet in the blood by specifically interacting with one or more sites in Env on the virion, thus they are expected to have higher utilization rate than the current antiviral drugs. The action mechanisms of PPVIs include (1) blocking the RBS on viral Envs, (2) inducing conformational changes of viral Env, causing the virion to lose the ability to enter the host cell, or (3) binding to the Env stem or the viral lipid membrane, to disrupt the integrity of the viral envelope or lead to the release of viral genetic materials. Apart from these, exact mechanisms of some PPVIs still remain to be explored.

In general, protein and peptide drugs are safer for humans than small-molecule chemical drugs because these big molecules do not enter the host cells, thus having no adverse effect on the functions of intracellular proteins. However, the effect of long-term use of PPVIs, especially those mimicking human proteins, such as the CD4 receptor, on the normal function of human body are still unknown, since many of these drug candidates have not been tested in clinics. To solve this problem, maybe we can design binding analogs of human proteins, like CD4M9 (Vita et al., 1999), or only select the core domain of natural structures, like mD1.22 mentioned above (Chen et al., 2014). Another potential problem is the immunogenicity of the protein- and peptide-based inactivators. After long-term use in humans, these exogenous proteins and peptides may induce specific antibodies against them, which may attenuate their inactivation activities. Therefore, immunogenicity of these drug candidates to humans should be assessed and reduced before further development. In recent years, strategies to remove the immunogenicity of the protein-based drug candidates are increasingly diverse and mature. In fact, they mainly focus on the T-cell epitope of the candidate, since T cell plays a key role in activating B cells to transform into antibody-producing plasma cells (Moise et al., 2016) and the linear T-cell epitope is more prone to predict than the steric B-cell epitope. One method adopted widely is to calculate the T-cell epitope with a forecasting software, such as Epimatrix developed by EpiVax Inc. (De Groot et al., 2008), validate the T-cell epitope with experiments (such as T-cell proliferation assay) and mutate its amino-acid sequence to remove the immunogenicity (Weber et al., 2009).

Besides these two disadvantages, compared with chemical-based virus inactivators, PPVIs usually have a shorter half-life and lack oral availability thus require several times of injection a week. In terms of their higher production cost, PPVIs are generally more expensive for long-term treatment of chronic viral infection. Lentiviral vector-based gene therapy to secret a PPVI continuously can be one choice to lower the cost (Perez et al., 2005; Egerer et al., 2011; Falkenhagen et al., 2014, 2017). For example, Falkenhagen et al. (2014) designed lentiviral vectors encoding secreted anti-HIV proteins including sCD4, which could prohibit the infection of both gene-modified and unmodified cells. They further investigated the *in vivo* application of this approach by injecting gene-modified hematopoietic stem/progenitor cells (HSPCs) into humanized mice. The results demonstrated a reduction of viral load over time in humanized mice capable of secreting sCD4, upon challenge with HIV (Falkenhagen et al., 2017). Therefore, continuous delivery of secreted PPVIs via gene therapy is also a potential way apart from oral administration of chemical-based virus inactivators or frequent injection of PPVIs.

In particular, the disadvantages of PPVIs mentioned above may not be the problems for the urgent treatment of the highly pathogenic emerging and re-emerging virus infections, e.g., Ebola virus or Middle East respiratory syndrome coronavirus (MERS-CoV) infection. Drugs are especially key in the first aid, when several days later, protective antibodies are produced in the body to combat viruses, but lives may be taken away at any time (Baseler et al., 2017; Mubarak et al., 2019). In fact, characteristics of medication against these viral infections are short-term use (1–3 weeks), rapid application and high safety for the infirm patients, therefore the cost may not be a problem to consider. Besides, the need of rapid application parallels to the injection requirement of PPVIs, which takes effect faster than oral administration. Also, protein-based drugs are intrinsically safe (Zaman et al., 2019), and in this circumstance, their short half-lives become an advantage instead because they will not accumulate in the body. In sum, PPVIs may be a good choice when facing the pandemic of the highly pathogenic newly emerging and re-emerging viruses.

Nowadays, a variety of protein and peptide drugs, including antibody drugs, have been approved for clinical use. Because of their advantages mentioned above, we believe that more and more PPVIs will be developed for treatment and prevention of viral infections, particularly useful for combating the pandemics or epidemics of newly emerging and re-emerging virus infections.

## Author Contributions

YW organized the manuscript. XS and QW collected related literatures, drew the table, and wrote the manuscript. LL revised the introduction, HIV, and influenza virus part of the manuscript. SJ revised the ZIKV and DENV, HSV, and the discussion part of the manuscript.

## Conflict of Interest

The authors declare that the research was conducted in the absence of any commercial or financial relationships that could be construed as a potential conflict of interest.
